# A Rare Case of Metformin-Induced Lactic Acidosis and Concomitant Euglycemic Ketoacidosis

**DOI:** 10.7759/cureus.73708

**Published:** 2024-11-14

**Authors:** Alex Shiplett, Jay Mathias

**Affiliations:** 1 Internal Medicine, Wright State University, Dayton, USA

**Keywords:** continuous renal replacement therapy (crrt), keto acidosis, metformin in dialysis patients, metformin-induced lactic acidosis, severe acidemia

## Abstract

Acidemia arises primarily from the accumulation of carbon dioxide or the loss of bicarbonate, leading to a pH decrease within the body, which can be fatal if severe and not promptly addressed. Metabolic acidemia occurs due to a loss of bicarbonate and can manifest through direct losses of bicarbonate via renal or gastrointestinal routes, or through the accumulation of anions such as lactic acid or ketoacids, leading to an anion gap metabolic acidosis. Many common etiologies for lactic acid and ketoacid generation exist, including medication-induced causes. Metformin-induced lactic acidemia is a well-known, yet rare, complication of metformin usage, but metformin-induced ketoacidemia is not well described. This report presents a case involving a patient with end-stage renal disease (ESRD) who, while taking metformin, developed severe anion gap metabolic acidosis with undetectably high levels of lactic acid and ketones, supporting the potential role of metformin in inducing severe lactic acidosis and ketoacidosis.

## Introduction

Acidemia is a common pathological state caused by an excess of protons in the blood, leading to a decreased blood pH level below 7.35 [1]. The Henderson-Hasselbalch equation demonstrates that the addition of carbon dioxide (CO2) or the loss of bicarbonate are the main mechanisms by which acid-base homeostasis is overwhelmed, leading to acidemia [2]. Clinically, acidemia is categorized as respiratory acidosis through the accumulation of CO2 due to poor ventilation, or metabolic acidosis through the loss of bicarbonate via direct renal or gastrointestinal losses. Metabolic acidosis can also occur through the accumulation of anions via various mechanisms, commonly referred to as anion gap metabolic acidosis (AGMA). Ketones are an example of anions that can accumulate in states where glucose is not readily available, serving as an alternative energy source. The generation of ketones most commonly occurs in diabetics in the form of diabetic ketoacidosis, but it has been recognized that certain diabetic medications can also produce abnormal amounts of ketones, leading to metabolic acidosis, such as the sodium-glucose cotransporter-2 inhibitors (SGLT2i). While there are reports of metformin causing severe ketone generation, they are not well described [3]. We present a case of a female patient with end-stage renal disease (ESRD) who was still taking metformin, and who presented with severe anion gap metabolic acidosis explained by undetectable high levels of lactic acid and ketones, to provide additional evidence that metformin can cause severe ketogenesis.

## Case presentation

**Figure 1:**
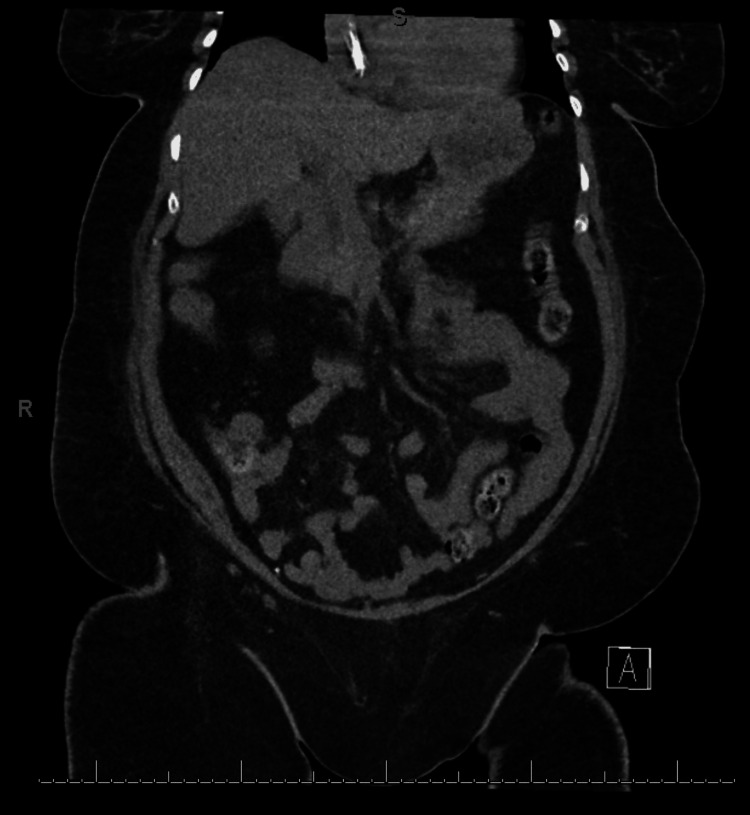
CT of the Abdomen and Pelvis Demonstrating No Acute Intra-abdominal Pathology CT - Computed Tomography

A 68-year-old female with a past medical history of morbid obesity, end-stage renal disease (ESRD) requiring hemodialysis three times a week, hypertension, hypothyroidism, and type 2 diabetes, presented to the emergency department after experiencing progressively worsening abdominal pain, nausea, and vomiting over the course of one day, having missed her dialysis session the day before. Initial lab work showed a severe metabolic acidosis with a pH of <6.8 on a venous blood gas. The patient’s bicarbonate was 3 mmol/L, with an anion gap of 46. To further assess the etiology of her anion gap, lactic acid and beta-hydroxybutyrate levels were obtained, yielding results of >20 mmol/L and >8 mmol/L, respectively. The differential diagnosis at this point included, but was not limited to, tissue ischemia, ketosis secondary to diabetic ketoacidosis, starvation or alcohol, toxic ingestion or exposure, and medication-induced causes. Interestingly, her blood glucose level was only slightly elevated at 121 mg/dL. She was not taking an SGLT2i due to her kidney disease, and she denied recent alcohol use, toxic ingestion, or any history suggestive of poor intake. Furthermore, the patient’s hemodynamics were normal, except for a heart rate between 110-120 beats per minute at the time of presentation to the intensive care unit. To evaluate possible causes of the severe lactic acidemia, given the reassuring hemodynamics, a computed tomography (CT) scan of the abdomen and pelvis was obtained to assess for bowel ischemia, but it revealed no acute intra-abdominal process (Figure 1).

With no obvious explanation for the lactic acidemia, a medication reconciliation was performed, revealing that the patient was taking metformin, which was considered the cause of her anion gap generation. The patient was started on a bicarbonate drip, insulin drip and continuous renal replacement therapy (CRRT) due to worsening hemodynamics. With these interventions, her lactic acid and ketoacid levels normalized by the end of the second day of hospitalization. Serial blood gases demonstrated that her arterial pH returned to 7.4 by the end of her first day in the hospital and her clinical status also improved over the next one to two days. The patient was subsequently educated to stop taking metformin due to her underlying kidney function and was discharged with a basal-bolus insulin regimen.

## Discussion

Metformin is a common first-line medication used for managing patients with type II diabetes (T2DM). It works by inhibiting hepatic gluconeogenesis and glycogenolysis [4]. Due to its efficacy and generally favorable safety profile, metformin is prescribed to over 120 million people worldwide [5]. Metformin is not metabolized in the body and is excreted unchanged in the urine. As a result of its pharmacokinetics, it is contraindicated in patients with severe renal dysfunction (defined as a glomerular filtration rate <30 ml/min), due to the increased risk of severe side effects from metformin accumulation. One well-described side effect of metformin is severe lactic acidemia through mitochondrial oxidative phosphorylation disruption, which occurs in 1 out of every 30,000 patients [6]. Although rare, metformin-induced lactic acidosis (MALA) carries a high mortality rate, with reports as high as 50% [7]. The recommended treatment involves dialyzing these patients acutely, alkalizing the body with bicarbonate, and ensuring they refrain from metformin use in the future [8]. Patients often recover with dialysis, as it removes metformin from the serum and replaces bicarbonate, improving acidosis and reversing life-threatening toxicity [9,10].

Ketoacidemia, characterized by elevated levels of ketone bodies in the blood, can arise from various causes. The most common causes include diabetic ketoacidosis (DKA), starvation ketosis, or alcoholic ketoacidosis. Ketoacids are produced when glucose uptake into cells is decreased, leading to increased lipolysis as an alternative energy source [11]. This process is common in conditions such as diabetes, alcoholism, and starvation, where impaired glucose utilization triggers the liver to generate ketone bodies from fatty acids. Diabetic ketoacidosis, marked by high blood glucose levels, is the most frequent cause, while euglycemic ketoacidosis occurs when ketoacidosis is present, but blood glucose remains below 250 mg/dL. Other causes of ketoacid production include medication-induced ketogenesis. The most common medications implicated are SGLT2 inhibitors, corticosteroids, and atypical antipsychotics [12]. Antipsychotics and corticosteroids increase insulin resistance leading to increased production of ketoacids, while SGLT2 inhibit the reabsorption of glucose in the proximal convoluted tubule, which in turn increases ketoacid reabsorption [13]. While not well described in the literature, metformin has also been shown to cause ketoacidemia. However, the pathophysiology remains poorly understood. One hypothesis suggests a mechanism resembling starvation ketoacidosis, where lack of oral intake leads to the body creating ketoacids for energy. This mechanism was considered in our case, as the patient presented with nausea and vomiting, although she reported eating earlier that day. Animal studies have also suggested a link between metformin use and increased fat oxidation and ketone body production, leading to ketoacidosis [7]. While this mechanism is one possible explanation, the exact pathway for metformin-induced ketone production in humans remains uncertain and exceedingly rare.

This patient presented with severe lactic acidosis and ketoacidosis, leading to a pH of <6.8, which is incompatible with life unless rapidly corrected. Severe acidemia is associated with a high mortality rate, and studies suggest that the rate of acidemia correction is a better indicator of prognosis than the initial pH [14]. Treatment for anion gap metabolic acidosis involves addressing the underlying cause. In this case, the cause was metformin toxicity in a patient with poor kidney function. Treatment included early intervention with bicarbonate infusion and CRRT, as the patient's hemodynamics worsened overnight. Enhanced metformin clearance is achievable with high-efficiency hemodialysis, while CRRT can also provide benefit in these patients, reducing mortality in cases where hemodialysis is not tolerated due to hemodynamic instability [10]. Managing metformin toxicity is challenging due to the severe acidosis, and the rarity of metformin-induced lactic acidosis and ketoacidosis often delays recognition. Early identification and prompt initiation of hemodialysis are crucial for managing these patients and preventing further complications. Therefore, a thorough medication reconciliation is vital in patient care, providing opportunities to adjust or discontinue medications as needed, especially in patients with kidney dysfunction, to prevent future ICU admissions and death.

## Conclusions

This case highlights the importance of considering metformin as a cause of severe acidemia in patients with new or worsening kidney dysfunction. The patient's presentation with severe lactic acidosis and euglycemic ketoacidosis underscores the potential life-threatening complications associated with metformin use in the context of impaired renal function. Early recognition and prompt intervention with renal replacement therapy were paramount to the patient's survival. This case also emphasizes the need for healthcare providers to conduct thorough medication reconciliations, particularly in patients with complex medical histories, to prevent similar life-threatening events. Educating patients about the risks associated with their medications, especially when there is a change in renal function, is essential for preventing future severe complications.
